# MicroRNAs MiR-218, MiR-125b, and Let-7g Predict Prognosis in Patients with Oral Cavity Squamous Cell Carcinoma

**DOI:** 10.1371/journal.pone.0102403

**Published:** 2014-07-22

**Authors:** Shih-Chi Peng, Chun-Ta Liao, Chien-Hua Peng, Ann-Joy Cheng, Shu-Jen Chen, Chung-Guei Huang, Wen-Ping Hsieh, Tzu-Chen Yen

**Affiliations:** 1 Department of Nuclear Medicine and Molecular Imaging Center, Chang Gung Memorial Hospital and Chang Gung University, Taoyuan, Taiwan, R.O.C.; 2 Department of Otorhinolaryngology, Head and Neck Surgery, Chang Gung Memorial Hospital and Chang Gung University, Taoyuan, Taiwan, R.O.C.; 3 Head and Neck Oncology Group, Chang Gung Memorial Hospital and Chang Gung University, Taoyuan, Taiwan, R.O.C.; 4 Resource Center for Clinical Research, Chang Gung Memorial Hospital and Chang Gung University, Taoyuan, Taiwan, R.O.C.; 5 Department of Medical Biotechnology, Chang Gung Memorial Hospital and Chang Gung University, Taoyuan, Taiwan, R.O.C.; 6 Department of Biomedical Sciences, Chang Gung Memorial Hospital and Chang Gung University, Taoyuan, Taiwan, R.O.C.; 7 Department of Pathology, Chang Gung Memorial Hospital and Chang Gung University, Taoyuan, Taiwan, R.O.C.; 8 Institute of Statistics, National Tsing Hua University, Hsinchu, Taiwan, R.O.C; The University of Hong Kong, China

## Abstract

MicroRNAs (miRNAs) have a major impact on regulatory networks in human carcinogenesis. In this study, we sought to investigate the prognostic significance of miRNAs in patients with oral cavity squamous cell carcinoma (OSCC). In a discovery phase, RNA was extracted from 58 OSCC tumor samples and paired normal tissues. MiRNAs expression was evaluated with TaqMan Array Card and TaqMan MicroRNA assays. The prognostic significance of the miRNA signature identified in the discovery phase was validated by qRT-PCR in a replication set consisting of 141 formalin-fixed, paraffin-embedded (FFPE) samples. We identified a miRNA regulatory network centered on the three hub genes (*SP1*, *MYC*, and *TP53*) that predicted distinct clinical endpoints. Three miRNAs (miR-218, miR-125b, and let-7g) and their downstream response genes had a concordant prognostic significance on disease-free survival and disease-specific survival rates. In addition, patients with a reduced expression of miR-218, miR-125b, and let-7g have a higher risk of poor outcomes in presence of specific risk factors (p-stage III-IV, pT3-4, or pN+). Our findings indicate that specific miRNAs have prognostic significance in OSCC patients and may improve prognostic stratification over traditional risk factors.

## Introduction

Oral cavity squamous cell carcinoma (OSCC) is a leading cause of cancer-related morbidity and mortality worldwide and has a significant societal and economic impact both in developed and developing countries [1]. The most established theory on the etiology of OSCC is one of an environmental trigger (e.g., smoking, alcohol, betel quid use, human papilloma virus infection) inducing a chronic inflammatory-proliferative response in the oral cavity of a genetically susceptible host. This evidence comes largely from epidemiologic and clinical studies of risk factors and genetic associations [2].

MicroRNAs (miRNAs) are small (20–24 nucleotides), evolutionarily conserved, non-coding RNAs that negatively regulate the translational efficiency and the stability of mRNAs. Over 1600 miRNAs are currently listed in the miRBase database [3], which are predicted to control the expression of at least 30% of all human genes [4]. Evidence garnered from experimental and clinical studies suggests that miRNAs play a major role in tumor biology, including apoptosis, cell cycle regulation, stress response, inflammation, cell proliferation, and invasiveness [5]. Some miRNAs can act as tumor suppressors whereas others behave as oncogenes [6]. Overexpression of oncogenic miRNAs may drive tumor initiation and progression, whereas the down-regulation of tumor-suppressing miRNAs can promote cancer development [7].

The study of miRNAs in OSCC is relatively scarce, however, some studies have reported the expression pattern of miRNAs in OSCC by high throughput technology and showed that deregulation of miRNAs affected normal cell growth and development, and even promote carcinogenesis [8], [9]. Several miRNAs (miR-34b, miR-100, miR-125b, miR-137, miR-193a, and miR-203) have been found to be significantly downregulated in OSCC samples [9]–[13]. In terms of the functional analysis, transfection of miR-125b and miR-100 to OSCC-derived cells significantly reduced cell proliferation in some studies [9], [14]. On the contrary, some miRNAs act as oncogenes, such as miR-221, miR-181, miR-375, miR-345 and miR-155 [9], [15]. Interestingly, the ratio of miR-221and miR-375 has been shown to predict OSCC with high sensitivity and specificity [16]. An increased expression of miR-21, miR-181b, and miR-345 has been associated with a higher risk of malignant transformation in patients with oral leukoplakias [17]. Moreover, a high expression of miR-211 has been shown to have an adverse prognostic impact in OSCC by promoting cyclin D1 overexpression [18].

There is evidence that miRNAs operate in complex regulatory networks [19], [20]. One miRNA may modulate the expression of tens-to-hundreds of different coding mRNAs. Conversely, a single mRNA may be simultaneously repressed by several distinct miRNAs [19], [21]. Most studies to date have focused on miRNA and mRNA expression profiles [20], [22], frequently in combination with sequence-based computational approaches to predict putative miRNA binding sites [23]. However, few studies have explored the downstream gene expression networks associated with altered miRNAs levels.

We have previously shown that OSCC is characterized by specific molecular prognostic signatures [24] and hypothesized that these molecular networks may be paralleled by specific miRNAs alterations. Herein, we describe the simultaneous assessment of the genome-wide expression profiles of both miRNAs (Data S1) and OSCC molecular signatures (Data S2) in paired tumor and normal samples. Based on qRT-PCR results and manually curated biological interaction maps, we were able to identify OSCC-specific miRNA regulatory networks that may modulate OSCC signatures and, in turn, significantly influence clinical outcomes.

## Materials and Methods

### Study subjects

Paired tumor specimens and adjacent non-tumor samples were obtained from 58 patients with biopsy-proven untreated primary OSCC. All of the samples were analyzed using the TaqMan Array Card and TaqMan MicroRNA Assay. Another independent set of OSCC tumor samples were collected from 141 patients with formalin-fixed, paraffin-embedded (FFPE) tissues. Patients who received radio- and/or chemotherapy before surgical sampling were excluded. The clinicopathological characteristics and the follow-up data were obtained from clinical charts. The study protocol was approved by the Ethics Committee of the Chang Gung Memorial Hospital (IRB approvals 100-1842A3 and 101–0171C). Written informed consent was obtained from all participants.

### Gene and miRNA expression profiling

We measured the mRNA levels of the previously identified 95 OSCC signatures [24], including 85 genes underlying variation in DNA copy number using real-time qPCR. Briefly, total RNA was extracted from fresh frozen tissues using the mirVana miRNA Isolation Kit (#AM1560, ABI). Reverse transcription was performed using the High Capacity cDNA RT Kit (#4368814, ABI) according to the manufacturer's protocol. For miRNA profiling study, total RNA was reverse transcribed using the TaqMan microRNA Reverse Transcription Kit (#4366596; ABI) with Megaplex RT primers (Human Pool A, #4399966; ABI; Human Pool B, # 4444281; ABI) and the RT products were analyzed in duplicate for gene expression using TaqMan Array Card Format 96b (# 4342261, ABI). For the validation study, miR-218, let-7g and miR-125b were quantified with the Single Tube TaqMan MicroRNA assay (ABI) following the manufacturer's protocol.

### Effects of M_4_N on OSCC cancer cell lines

Three OSCC cancer cell lines (OECM1, CG-C10, and SAS) were selected for *in vitro* studies. Cell origins and culture conditions for maintenance have been previously described [25]. Tetra-*O*-methyl nordihydroguaiaretic acid (M_4_N; 40 µM for 4 days) was used as a global transcription inhibitor [26] as previously described [27]. After harvesting the cells, miRNAs were extracted and measured using TaqMan miRNAs assays kits (ABI, Foster City, CA, USA). The expression levels of miR-125b, miR-leg-7g, and miR-128 were determined by real-time qPCR method using specific primers [25].

### Statistical analysis

We used the Wilcoxon signed rank test to compare the expression levels of miRNAs in paired tumor and control specimens. The false discovery rate (FDR) procedure was used to adjust for multiple testing. The miRNA expression values were dichotomized according to the median for survival analysis. Local control, neck control, distant metastases, disease-free survival, disease-specific survival, and overall survival served as the main outcome measures. Survival curves were plotted using the Kaplan-Meier method (log-rank test). Logistic regression analysis was used to identify the associations between miRNA patterns, downstream genes, and clinical outcomes.

### Associations of miRNA expression with prognostic gene clusters

**Figure 1:**
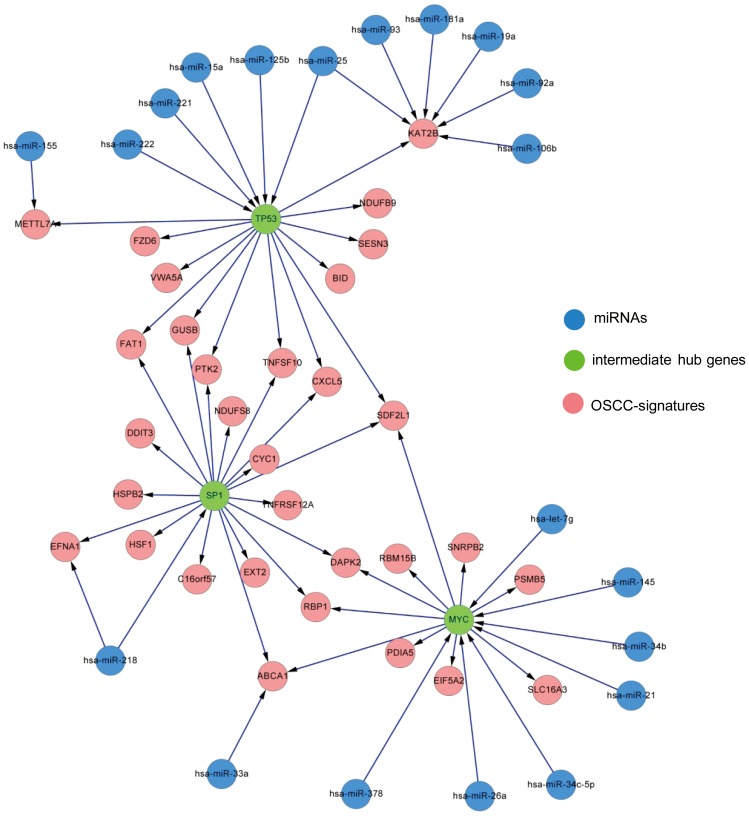
OSCC-specific microRNA regulatory network centered on the three hub genes (*SP1*, *MYC*, and *TP53*; green nodes). The red nodes indicate the OSCC signatures identified in our previous study [24]. The blue nodes represent the miRNAs that were differentially expressed in OSCC samples. The edges from the miRNAs to the different targets indicate the experimentally-confirmed relationships according to the miRTarBase and TarBase databases. The edges between the intermediate hub genes and the OSCC signatures represent the known molecular interactions or the canonical pathways which were manually curated in the MetaCore database.

We used sparse partial least squares regression (SPLS) to investigate the associations between the expression of miRNAs upstream to the three hub genes and the expression of outcome-specific gene clusters. SPLS is a variable selection technique for multivariate responses [28]. The expression profiles of each outcome-specific gene cluster were considered as the dependent variables, whereas all of the significant miRNAs identified in the regulatory network (Figure 1) were entered as potential predictors. The details of the modeling are summarized in the Method S1. By using this methodology, we identified the expression of miRNAs affecting the downstream outcome-specific gene clusters.

## Results

### miRNA regulatory network of OSCC signatures

In this study, we sought to understand the regulation map of miRNAs and to identify their prognostic significance in OSCC. We initially built a regulatory network to connect the miRNAs found to be dysregulated in OSCC with the prognostic mRNAs which have been previously associated with prognosis [24]. Using a significance threshold of p<0.001 in Wilcoxon rank-sum tests, 232 of the 760 miRNAs assessed on the TaqMan Array Card were found to be differentially expressed between paired tumor and normal tissue samples. The potential regulation path from the identified miRNAs to the 95 OSCC-signatures was constructed based on both the binding information and interaction map from public databases.

According to the miRTarBase [29] and TarBase [30] databanks, there were hundreds of genes that were directly regulated by the 232 miRNAs in our initial list. To narrow our search to clinically relevant genes, only the genes annotated as cancer-related genes in the KEGG database (which collects most of the cancer pathways and the main pharmacological targets of approved cancer drugs) were considered. The focus was thus restricted to the 84 genes that were direct targets of the 89 miRNAs.

The 84 selected genes served as molecular proxies between the observed miRNAs alterations and the previously identified OSCC signatures [24]. The associations between the 84 genes and the OSCC signatures were identified using a collection of confirmed molecular interactions and pathways in the MetaCore suite (GeneGo Inc., St. Joseph, MI, USA). We used the shortest path routing algorithm for the purpose of analysis. We finally reached a miRNA regulatory network that contains 49 miRNAs, 39 intermediate genes and 45 OSCC-signatures. It is noteworthy that only three intermediate genes (*SP1*, *TP53*, *MYC*) showed at least 10 degrees of connection, suggesting that each of them regulated at least 10 distinct OSCC signatures. The three intermediate genes were found to control relatively more OSCC signatures in our network than other genes and were thus considered as hub regulatory genes. We then extracted a SP1-TP53-MYC centered subnetwork (Figure 1). Because *SP1*, *TP53*, and *MYC* play a well-known role in carcinogenesis, we specifically examined the associations of their upstream miRNAs with the clinical outcomes.

### Association of miRNAs with clinical outcomes

We then investigated the prognostic significance of the miRNAs that specifically bind to the hub regulatory genes (*SP1*, *TP53*, and *MYC*) with clinical outcomes. After variable selection in multivariable logistic regression analysis, we found that miR-218 was the only miRNA (Figure 1) that regulated *SP1* and was a significant predictor for disease-free survival (odds ratio [OR]  = 3.215, p = 0.037) and disease-specific survival (OR = 3.012, p = 0.049) (Table S1). Of the miRNAs regulating *MYC*, let-7g was associated with local control (OR = 5.917, p = 0.032), neck control (OR = 250, p = 0.009), distant metastases (OR = 31.25, p = 0.023), disease-free survival (OR = 6.329, p = 0.002), and disease-specific survival (OR = 6.329, p = 0.004). In addition, the association of neck control with miR-33a (OR = 11.827, p = 0.025) and miR-378 (OR = 8.403, p = 0.034) were also statistically significant (Table S2). Of the miRNAs regulating *TP53*, miR-125b, also the only miRNA has shown significant predicton of neck control (OR = 5.015, p = 0.054), disease-free survival (OR = 4.444, p = 0.01), disease-specific survival (OR = 4.651, p = 0.032), and overall survival (OR = 4.464, p = 0.023) (Table S3).

### Association between downstream OSCC signatures and clinical outcomes

Using multivariable logistic regression analysis, we subsequently analyzed the associations between the clinical outcomes and the downstream signatures controlled by the three hub regulatory genes (Tables S4–S6). The OSCC signatures regulated by *SP1* (*EXT2*, *FAT1*, and *ABCA1*) were associated with disease-free survival, disease-specific survival, and overall survival. *MYC*-associated signatures (*ABCA1* and *PDIA5*) were predictors of disease-free survival, disease-specific survival, and poor survival. *TP53*-related signatures (*TNFSF10* and *NDUFB9*) were associated with disease-free survival and disease-specific survival rates.

The OSCC signatures targeted by the same hub gene and associated with the same clinical outcomes were grouped together into an outcome-specific gene cluster. The details of outcome-specific gene clusters are presented in Tables S4–S6. The miRNAs and signatures that act upstream and downstream of *SP1* were associated with disease-free survival and disease-specific survival, The signatures acting upstream and downstream of *MYC* were associated with neck control, distant metastasis, disease-free survival, and disease-specific survival, Finally, the miRNAs and signatures associated with *TP53* predicted neck control, disease-free survival, disease-specific survival, and overall survival. Taken together, these results demonstrate that the prognostic impact of OSCC signatures is concordant with the observed changes in the expression of the corresponding upstream miRNAs.

### MiRNAs as prognostic modulators

**Figure 2:**
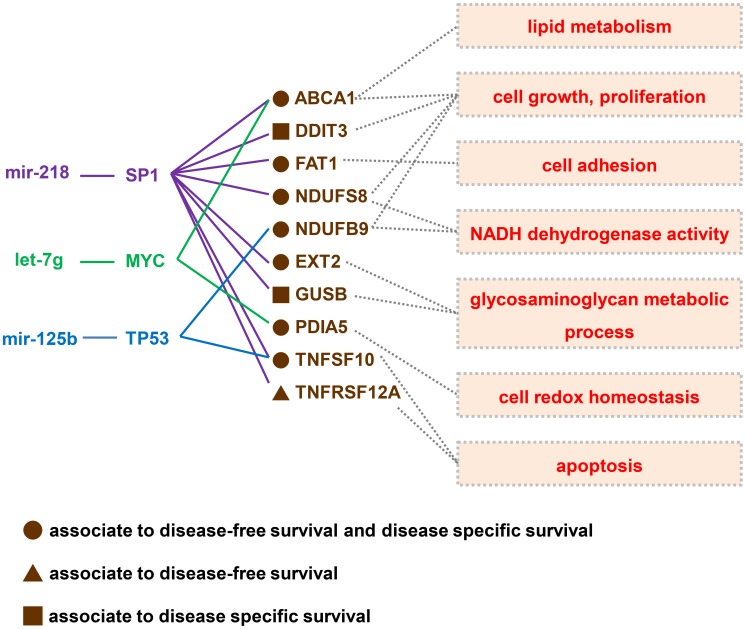
Prognostic miRNA modulators centered on the three hub genes *SP1*, *MYC*, and *TP53*. The downstream genes were grouped into outcome-specific clusters according to their prognostic significance (Tables S4–S6). The associations between the upstream miRNAs and the downstream gene clusters were identified using sparse partial least square regression. Both the upstream miRNAs and the downstream responsive gene clusters showed a concordant prognostic impact on disease-free survival and disease-specific survival. The solid lines indicate the experimentally-confirmed physical interactions. The dotted lines show the results of the functional analysis performed using the DAVID package. *ABCA1*, *DDIT3*, *NDUFS8*, and *NDUFB9* regulate cell growth and proliferation. *TNFSF10* and *TNFRSF12A* are involved in the cell's apoptotic machinery. The remaining genes encode for molecules regulating cell adhesion or glycosaminoglycan metabolism. The miRNA modulators identified in this predicted poor outcomes in OSCC. Hopefully, our findings may lead to the development of novel prognostic models integrating molecular signatures and traditional risk factors for improving the prognostic stratification and the treatment modalities of OSCC patients.

A prognostic modulator was defined as a set consisting of the upstream miRNAs and the corresponding downstream genes associated with a given clinical outcome. We hypothesized that the miRNAs and OSCC signatures could influence prognosis through common molecular pathways. We therefore analyzed whether the expression of the OSCC signatures driven by the three hub regulatory genes (*SP1*, *TP53*, and *MYC*) was correlated with the expression of the miRNAs potentially controlling the hub genes. We used sparse partial least squares (SPLS) regression [31] to identify the miRNAs that may influence the expression of OSCC signatures located downstream to the three hub genes. Because we grouped the OSCC signatures into distinct outcome-specific gene clusters with the associated signatures for *SP1* (Table S4), *MYC* (Table S5) and *TP53* (Table S6), the expression profile of each cluster was analyzed in a multivariable manner using SPLS analysis. All of the significant miRNAs shown in Figure 1 were entered into the model as potential predictors. As expected, the specific upstream miRNAs (miR-218, let-7g, and miR-125b) were selected by the model as corresponding to the downstream target gene clusters. MiR-218 correlated with the expression of two *SP1*-related gene clusters related to disease-free survival and disease-specific survival. Let-7g was associated with the expression of a *MYC*-related gene cluster, which had an adverse impact on disease-free survival and disease-specific survival. Finally, the expression of miR-125b was found to be related to the expression of *TP53*-related gene clusters associated with disease-free survival and disease-specific survival. The miRNA prognostic modulators centered on the three hub regulatory genes are depicted in Figure 2. The upstream regulatory miRNAs (miR-218, let-7g, and miR-125b) and the related downstream gene clusters were found to have a concordant prognostic value on disease-free survival and disease-specific survival. Most of the downstream OSCC signatures (e.g., *EXT2*, *NDUFS8*, *TNFSF10*, *FAT1*, *ABCA1*, *PDIA5*, and *NDUFB9*) were associated with both clinical outcomes simultaneously. Therefore, the genes involved in disease-free survival may also influence overall survival. We then annotated the OSCC signatures using the functional annotation tool of the DAVID package [32] (Figure 2). *ABCA1*, *DDIT3*, *NDUFS8*, and *NDUFB9* are involved in cell growth and proliferation. *TNFSF10* and *TNFRSF12A* play an important role in the regulation of apoptosis. *EXT2* and *GUSB* are involved in glycosaminoglycan metabolism, whereas *FAT1* plays a role in cell adhesion. Taken together, these data suggest that miR-218, let-7g, and miR-125b may be valuable prognostic indicators of disease-free survival and disease specific survival.

To validate the clinical significance of the miRNA signature, we carried out qRT-PCR for miR-218, let-7g and miR-125b in an independent cohort. As expected, the three miRNA had significant association with disease-free survival and disease specific survival (p<0.05, Table S7). One unit decrease in the expression level of miR-218 and miR-125b increased the risk of disease recurrence and death by approximately two-fold. Concerning let-7g, one unit decrease in its expression increased the risk of disease recurrence by 2.6-fold and, most significantly, increased the risk of death by 12.9-fold.

### Clinical impact of the prognostic modulators in risk stratification

**Figure 3:**
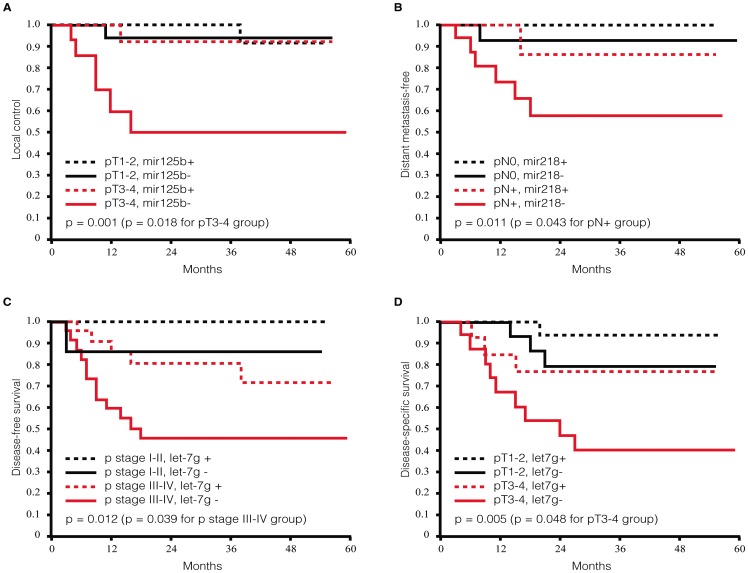
Kaplan-Meier survival plots of OSCC patients according to traditional risk factors and miRNAs expression levels. (**A**) In the subgroup of patients with pT3-4 disease, subjects with high and low miR-125b expression had significantly different local control rates (92% vs. 50%); (**B**) the subgroup with low miR-218 expression showed a higher rate of distant metastases in patients with pN+ disease (86% vs. 57%); (**C**) a high let-7g expression was associated with a lower risk of tumor relapse in patients with advanced pathological stage (46% vs.71%); (**D**) an increased expression of let-7g predicted a better disease-specific survival in the subgroup of patients with pT3-4 disease (76% vs. 40%).

We next investigated whether the three miRNAs and the ten downstream OSCC signatures (Figure 2) may improve risk stratification beyond the information provided by traditional risk factors (pathological stage, pathological tumor status, pathological nodal status, and tumor differentiation). The expression of each miRNA was dichotomized according to the median value and compared using the log-rank test. Advanced tumor status (pT3-4) is a risk factor for local control and disease-specific survival [33], whereas pathological lymph node metastases predict distant metastases [34]. Moreover, the pathological stage is associated with the risk of poor disease-free survival [33]. Multivariable Cox regression analysis demonstrated that the three miRNAs improved the prognostic stratification of OSCC patients and had a higher predictive than the downstream signatures on the clinical outcomes. Table 1 summarizes the prognostic significance of the miRNAs in combination with traditional risk factors. For the purpose of analysis, we dichotomized the clinicopathological characteristics of the participants as follows: pathological tumor status (pT1-2 vs. pT3-4), pathological nodal status (pN0 vs. pN+), pathological stage (p-stage I-II vs. p-stage III–IV), and tumor differentiation (well/moderate vs. poor). The combination between traditional risk factors and the expression of the three miRNAs led to identification of specific high-risk groups (Figure 3). In patients with pT3-4 disease, the expression of miR-125b had a significant impact on local control (p = 0.018, Figure 3A). In patients with pN+, a low miR-218 expression was associated with an increased risk of distant metastases (p = 0.043, Figure 3B). An increased expression of let-7g was associated with a lower incidence of poor disease-free survival in patients with advanced pathological stages (p = 0.039, Figure 3C). Notably, a higher expression of let-7g was also associated with better disease-specific survival rates in patients with pT3-4 disease (p = 0.048, Figure 3D).

**Table 1:** Multivariable analysis of 5-year control and survival rates in OSCC patients (*n* = 58).

	Local control	Neck control	Distant metastases	Disease-free survival	Disease-specific survival	Overall survival
Risk factors (*n*)	*p*, HR (95%CI)	*p*, HR (95%CI)	*p*, HR (95%CI)	*p*, HR (95%CI)	*p*, HR (95%CI)	*p*, HR (95%CI)
pT3-4 (*n* = 28)	0.026*, 6.152 (1.248, 30.336)	ns	ns	ns*	0.012*, 4.317 (1.388, 13.429)	ns*
pN+ (*n* = 35)	ns	ns	0.046*, 8.411 (1.043, 67.835)	ns	ns*	ns*
p-stage III–IV (*n* = 43)	ns*	ns	ns*	0.052*, 7.415 (0.982, 55.963)	ns	ns
Poor differentiation (*n* = 7)	ns	ns	ns	ns	ns	ns
miR-218 (*n* = 29)	ns	ns	0.048, 4.90 (1.012, 23.809)	ns*	ns*	ns
Let-7g (*n* = 29)	ns*	ns*	ns	0.025*, 3.267 (1.164, 9.174)	0.039*, 3.289 (1.059, 10.204)	ns
miR-125b (*n* = 29)	0.046*, 4.694 (0.959, 22.727)	ns*	ns	ns*	ns*	ns*

Abbreviations: HR, hazard ratio; CI, confidence interval; ns, not significant.

* Indicates risk factors significantly associated with outcomes in univariate analysis.

In another set of experiments, we have then tested the association between the three miRNA signatures and clinical outcomes in another validation panel of patients with pT3-4 disease, pN+, or advanced pathological stages. For example, we found in patients with pT3-4 disease, the decrease in one unit of miR-125b expression increased the risk of local recurrence by 9.5-fold (p = 0.048). As for patients with pN+, the decrease in one unit of miR-218 expression also increased the risk of distant metastasis by 2.1-fold (p = 0.009). The expression of let-7g was marginally associated with disease-free survival in patients with advanced pathological stages (p = 0.053) and disease-specific survival rates in patients with pT3-4 disease (p = 0.071). Taken together, our results indicate that the integration of the traditional risk factors and the miRNA signature may improve the prognostic stratification of OSCC patients.

### Effect of global transcription inhibition on OSCC cell growth and mir-218, let-7g, and mir-125b expression

**Figure 4:**
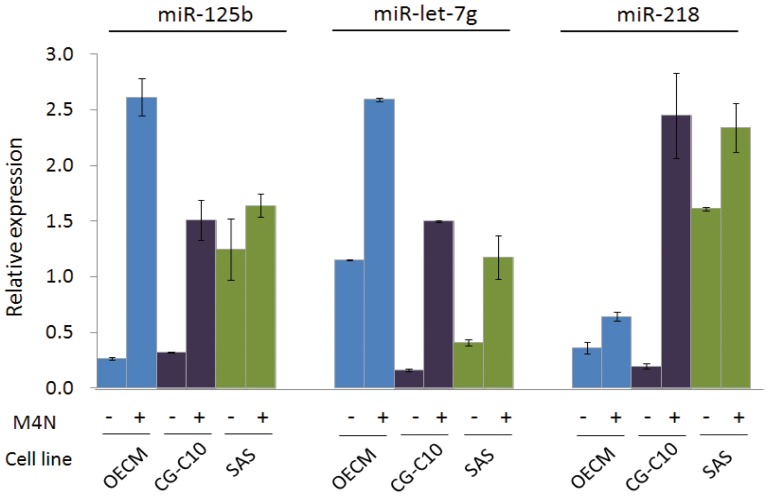
Effects of M_4_N on the expression of miR-218, miR- let-7g, and miR-125b in OSCC cancer cell lines. We used three OSCC cancer cell lines (OECM, CG-C10, and SAS) in the experiments. After treatment with M_4_N (40 µM) for two days, cells were harvested and subjected to miRNA expression analysis using the RT-qPCR method. For each miRNA, the expression levels recorded with threshold cycle numbers (Ct) were normalized against an internal control (U6 RNA). The comparative threshold cycle method (ΔCt) was used to quantify the relative miRNA expression.

Because the prognostic signature of three miRNAs, mir-218, let-7g, and mir-125b were found to be the major miRNAs that associated with the three hub genes (*SP1*, *MYC*, and *TP53*) predicted to be their targets, we sought to determine whether there were concordant changes in their expression after *in vitro* treatment with the global transcription inhibitor tetra-*O*-methyl nordihydroguaiaretic acid (M_4_N) [26] To this aim, we exposed cell lines derived from OSCC patients to M_4_N and evaluated the cultured cells for proliferation, death, and the expression of the three hub genes with their potential miRNA regulators. In general, a short exposure to M_4_N resulted in a significant inhibition of cell growth, whereas longer treatments caused cell death (Figure S1). While after exposure to M_4_N, the expression of mir-218, let-7g, and mir-125b was significantly increased in all of the cell lines (Figure 4). These results lend support to a modulatory role for the three miRNAs on the three hub genes.

## Discussion

In this study, we examined the associations between key regulatory miRNAs, molecular signatures, and clinical outcomes in OSCC patients. Notably, we identified three miRNAs (miR-218, let-7g, and miR-125b) that play a key role as prognostic modulators in OSCC patients. Our findings have important translational implications. In patients with traditional risk factors, we were able to identify distinct prognostic subgroups based on the expression of miR-218, miR-125b, and let-7g. Such expression changes can reflect distinct biological subtypes that may in turn be associated with different disease trajectories. Taken together, our pilot results may open new avenues focusing on miRNAs as prognostic biomarkers and therapeutic targets in OSCC.

Recent studies have shown that miR-218 and let-7g can inhibit cell invasion and metastasis in gastric cancer [35] and breast cancer [36], respectively. Moreover, miR-125b has been found to be dysregulated in ovarian cancer [37], breast cancer [38], and prostate cancer [39]. In OSCC, miR-125b also has been found to be downregulated [13] and showed a significant negative correlation with TP53 [40]. These data are in accordance with our results showing that an increased expression of mir-218, let-7g, and mir-125b predicts disease-free and disease-specific survival in OSCC patients (Figure 3).

We also demonstrated that the OSCC signature clusters located downstream to the three miRNAs were highly overlapping and were modulated by three hub genes (*SP1*, *MYC*, and *TP53*; Figure 2). Such transcriptional regulations have been experimentally confirmed by *in vitro* experiments using different approaches (Table S8 and References). The targets of *TP53*, such as *NDUFB9*, were identified using chromatin immunoprecipitation (ChIP) coupled with paired end ditag (PET) analysis to enhance mapping accuracy and sequencing efficiency. The same ChIP-PET approach was used to identify the target of *MYC*, i.e. *PDIA5*. Other transcriptional interactions (such as *TP53*-*TNFSF10*, *SP1*-*NDUFS8*, *SP1*-*ABCA1*, and *SP1-TNFSF10*) were analyzed using the transient transfection reporter assay. The electrophoretic mobility shift assay (EMSA) was also used to confirm the ability of the binding elements to interact with the cognate transcription factors. Both the upstream miRNAs and the downstream responsive gene clusters were associated with disease-free survival and disease-specific survival rates in OSCC patients. Therefore, we hypothesize that the three key miRNAs identified in our study may act in a coordinate fashion to influence the biological behavior of OSCC. Importantly, they may ultimately have a major impact on clinical outcomes through the downstream OSCC signature genes.

Functional analyses of the downstream signatures identified have shown that such genes are involved in cell proliferation, cell adhesion, metabolism of glycosaminoglycans, and apoptosis in a variety of cancers. *ABCA1* has been found to promote cell proliferation in human androgen-dependent prostate cancer LNCaP cells [41]. *EXT2* encodes for a glycosyltransferase that catalyzes the polymerization of heparan sulfate. Notably, glycosaminoglycans play a crucial role in several pathways involved in the control of cell growth and proliferation, including the Hedgehog and Wnt signaling cascades [42]. Mutations in *EXT2* have been also reported in patients with multiple osteochondromas [43]. *NDUFS8* and *NDUFB9* encode for two proteins embedded in the inner mitochondrial membrane and involved in the respiratory electron transport chain. The overexpression of *NDUFS8* and *NDUFB9* may enhance cell proliferation by promoting efficient energy metabolism [44]. Interestingly, *NDUFS8* has been associated with tumor relapse in patients with estrogen receptor α-positive breast cancer [45]. An increased expression of *NDUFB9* also imparts a higher risk of lymph node metastasis in esophageal squamous cell carcinoma [46]. *TNFSF10* (*TRAIL*) and *TNFRSF12A* are involved in the cell's apoptotic machinery. *TNFSF10* can induce apoptosis in several cancer cell lines with minimal toxicity against normal cells [47]. In oral cancer cells, cathepsin B has been shown to mediate *TNFSF10*-induced apoptosis [48]. *TNFRSF12A* is a transmembrane receptor that plays an essential role in cell proliferation, invasion, and migration of androgen-independent prostate cancer cells through its binding to the multifunctional cytokine tumor necrosis factor-like weak inducer of apoptosis (*TWEAK*) [49]. Although the cadherin gene *FAT1* is likely to function as a tumor suppressor in OSCC [50], silencing its expression has been shown to exert only limited effects on the proliferation of OSCC cell lines [51]. Therefore, further studies are needed to shed more light on the potential role of *FAT1* in the biology of OSCC.

From a translational standpoint, the results of our study reveal that the integration of the expression of the miR-218, let-7g, and miR-125b with traditional risk factors may improve current stratification strategies. Our findings demonstrate that OSCC patients with pN+ and lower expression of miR-218 have a dismal distant metastatic rate. In subjects with pT3-4 disease, those with a lower miR-125b expression had an increased risk of poor local control. Moreover, a low level of let-7g expression identified a high-risk subgroup of pT3-4 patients with poor disease-specific survival. Among patients with p-stage III–IV, a decreased expression of let-7g resulted in a reduced disease-free survival. Consequently, high-risk patients should avoid unnecessary interventions with curative intent or may be treated with alternative novel therapies. By contrast, a high expression of miR-218, let-7g, or miR-125b is associated with better clinical outcomes in patients' adverse pathological risk factors (pT3-4, pN+, and p-stage III–IV). Therefore, the study of miRNAs expression may be clinically useful for tailoring treatment strategies and optimizing follow-up protocols. A large prospective study is recommended to further validate the prognostic impact of the miRNAs identified in the current study.

In summary, we identified three miRNAs that may serve as key prognostic indicators in OSCC through their influence on downstream OSCC signatures. Hopefully, our findings may lead to the development of novel prognostic models integrating molecular signatures and traditional risk factors for improving the prognostic stratification and the treatment modalities of OSCC patients.

## Supporting Information

Figure S1 **Effects of M_4_N on the growth of OECM (A) and SAS (B) oral squamous cell carcinoma cell lines.** After treatment with M_4_N (40 µM), the number of cells was counted every two days.
(DOC)

Table S1 **Logistic regression analysis of clinical outcomes independently associated with the miRNAs binding to SP1.**
(DOC)

Table S2 **Logistic regression analysis of clinical outcomes independently associated with the miRNAs binding to MYC.**
(DOC)

Table S3 **Logistic regression analysis of clinical outcomes independently associated with the miRNAs binding to TP53.**
(DOC)

Table S4 **Logistic regression analysis of clinical outcomes independently associated with the SP1-associated signatures.**
(DOC)

Table S5 **Logistic regression analysis of clinical outcomes independently associated with the MYC-associated signatures.**
(DOC)

Table S6 **Logistic regression analysis of clinical outcomes independently associated with the TP53-associated signatures.**
(DOC)

Table S7 **Logistic regression analysis of clinical outcomes associated with the miR-218, let-7g, and miR-125b in validation cohort.**
(DOC)

Table S8 **Evidence derived from previous studies on the interactions between the network edges of the miRNA modulators.**
(DOC)

Method S1 **Description of the sparse partial least squares regression.**
(DOC)

Data S1 **miRNA_expression.** (dataS1_miRNA.xlsx).
(XLSX)

Data S2 **95 genes_expression.** (dataS2_mRNA.xlsx).
(XLSX)
