# Supplementary material for: MicroRNAs MiR-218, MiR-125b, and Let-7g Predict Prognosis in Patients with Oral Cavity Squamous Cell Carcinoma
Source: PLoS One. 2014 Jul 22;9(7):e102403. doi: 10.1371/journal.pone.0102403 (PMC4106832; doi:10.1371/journal.pone.0102403)
Supplement: Figure S1 — Effects of M4N on the growth of OECM (A) and SAS (B) oral squamous cell carcinoma cell lines. After treatment with M4N (40 µM), the number of cells was counted every two days. (DOC) [file pone.0102403.s001.doc]

**
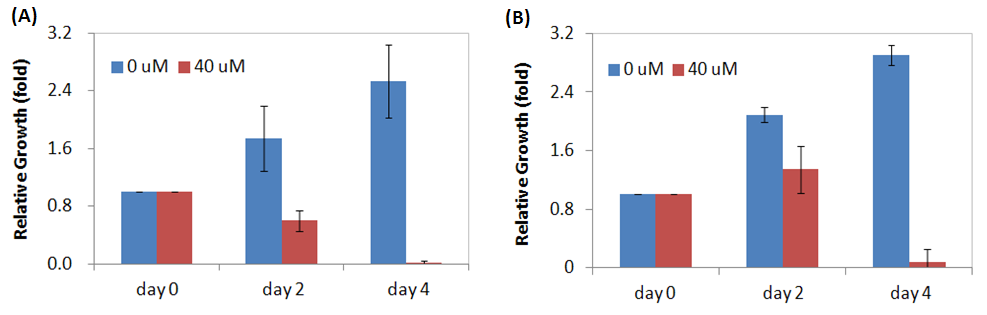
**

**Figure S1.** **Effects of M_4_N on the growth of OECM (A) and SAS (B) oral squamous cell carcinoma cell lines.** After treatment with M_4_N (40 µM), the number of cells was counted every two days.
