# Supplementary material for: MicroRNAs MiR-218, MiR-125b, and Let-7g Predict Prognosis in Patients with Oral Cavity Squamous Cell Carcinoma
Source: PLoS One. 2014 Jul 22;9(7):e102403. doi: 10.1371/journal.pone.0102403 (PMC4106832; doi:10.1371/journal.pone.0102403)
Supplement: Table S1 — Logistic regression analysis of clinical outcomes independently associated with the miRNAs binding to SP1. (DOC) [file pone.0102403.s002.doc]

**Table S1** Logistic regression analysis of clinical outcomes associated with the miRNAs binding to *SP1*

| **Event** | **Predictor** | **P value** | **Odds ratio (95%CI)** |
| --- | --- | --- | --- |
| Disease-free survival | has-mir-218 | 0.037 | 3.215 (1.071, 9.615) |
| Disease-specific survival | has-mir-218 | 0.049 | 3.012 (1.106, 9.433) |
