# Supplementary material for: MicroRNAs MiR-218, MiR-125b, and Let-7g Predict Prognosis in Patients with Oral Cavity Squamous Cell Carcinoma
Source: PLoS One. 2014 Jul 22;9(7):e102403. doi: 10.1371/journal.pone.0102403 (PMC4106832; doi:10.1371/journal.pone.0102403)
Supplement: Table S2 — Logistic regression analysis of clinical outcomes independently associated with the miRNAs binding to MYC. (DOC) [file pone.0102403.s003.doc]

**Table S2** Logistic regression analysis of clinical outcomes associated with the miRNAs binding to *MYC*

| **Event** | **Predictor** | **P value** | **Odds ratio (95%CI)** |
| --- | --- | --- | --- |
| Local control | hsa-let-7g | 0.032 | 5.917 (1.163, 15.873) |
| Neck control | hsa-let-7g  hsa-miR-33a  hsa-miR-378 | 0.009  0.025  0.034 | 250 (40, 1000)  11.827 (3.358, 22.990)  8.403 (1.174, 58.824) |
| Distant metastasis | hsa-let-7g | 0.023 | 31.250 (3.717, 62.500) |
| Disease-free survival | hsa-let-7g | 0.002 | 6.329 (1.919, 20.833) |
| Disease-specific survival | hsa-let-7g | 0.004 | 6.329 (1.786, 22.222) |
