# Supplementary material for: MicroRNAs MiR-218, MiR-125b, and Let-7g Predict Prognosis in Patients with Oral Cavity Squamous Cell Carcinoma
Source: PLoS One. 2014 Jul 22;9(7):e102403. doi: 10.1371/journal.pone.0102403 (PMC4106832; doi:10.1371/journal.pone.0102403)
Supplement: Table S3 — Logistic regression analysis of clinical outcomes independently associated with the miRNAs binding to TP53. (DOC) [file pone.0102403.s004.doc]

**Table S3** Logistic regression analysis of clinical outcomes associated with the miRNAs binding to *TP53*

| **Event** | **Predictor** | **P value** | **Odds ratio (95%CI)** |
| --- | --- | --- | --- |
| Neck control | hsa-miR-125b | 0.054 | 5.051 (1.916, 26.316) |
| Disease-free survival | hsa-miR-125b | 0.01 | 4.444 (1.996, 13.889) |
| Disease-specific survival | hsa-miR-125b | 0.032 | 4.651 (2.646, 12.048) |
| Overall survival | hsa-miR-125b | 0.023 | 4.464 (2.392, 16.129) |
