# Supplementary material for: MicroRNAs MiR-218, MiR-125b, and Let-7g Predict Prognosis in Patients with Oral Cavity Squamous Cell Carcinoma
Source: PLoS One. 2014 Jul 22;9(7):e102403. doi: 10.1371/journal.pone.0102403 (PMC4106832; doi:10.1371/journal.pone.0102403)
Supplement: Table S4 — Logistic regression analysis of clinical outcomes independently associated with the SP1-associated signatures. (DOC) [file pone.0102403.s005.doc]

**Table S4** Logistic regression analysis of clinical outcomes associated with the *SP1*-associated signatures

| **Event** | **Signature** | **P value** | **Odds ratio (95%CI)** |
| --- | --- | --- | --- |
| Disease-free survival | *EXT2*  *NDUFS8*  *TNFSF10*  *FAT1*  *ABCA1*  *TNFRSF12A* | 0.014  0.041  0.006  0.031  0.041  0.056 | 0.393 (0.187, 0.826)  0.509 (0.225, 0.855)  3.460 (1.437, 8.333)  0.554 (0.324, 0.949)  1.767 (1.114, 3.597)  1.481 (1.230, 2.445) |
| Distant metastasis | *FAT1*  *ABCA1* | 0.049  0.025 | 0.512 (0.248, 0.671)  2.747 (1.139, 6.623) |
| Disease-specific survival | *EXT2*  *NDUFS8*  *DDIT3*  *FAT1*  *ABCA1*  *GUSB* | 0.046  0.024  0.016  0.014  0.011  0.014 | 0.456 (0.191, 0.520)  0.279 (0.092, 0.843)  0.367 (0.163, 0.827)  0.356 (0.156, 0.813)  3.436 (1.333, 8.850)  3.215 (1.264, 8.197) |
| Overall survival | *EXT2*  *HSPB2*  *DDIT3*  *EFNA1*  *FAT1*  *ABCA1*  *HSF1*  *DAPK2*  *TNFRSF12A*  *C16orf57*  *GUSB* | 0.008  0.042  0.021  0.014  0.030  0.013  0.012  0.034  0.049  0.021  0.019 | 16.924 (2.116, 135.389)  1.969 (1.027, 3.788)  7.866 (1.369, 45.203)  3.861 (1.314, 11.364)  3.842 (1.143, 12.912)  12.658 (1.698, 90.909)  5.249 (1.445, 19.067)  5.443(1.138, 26.038)  2.326 (1.003, 5.405)  5.556 (1.292, 23.810)  19.608 (1.631, 250) |
