# Supplementary material for: MicroRNAs MiR-218, MiR-125b, and Let-7g Predict Prognosis in Patients with Oral Cavity Squamous Cell Carcinoma
Source: PLoS One. 2014 Jul 22;9(7):e102403. doi: 10.1371/journal.pone.0102403 (PMC4106832; doi:10.1371/journal.pone.0102403)
Supplement: Table S5 — Logistic regression analysis of clinical outcomes independently associated with the MYC-associated signatures. (DOC) [file pone.0102403.s006.doc]

**Table S5** Logistic regression analysis of clinical outcomes associated with the *MYC*-associated signatures

| **Event** | **Signature** | **P value** | **Odds ratio (95%CI)** |
| --- | --- | --- | --- |
| Neck control | *RBM15B* | 0.011 | 1.451 (1.089, 1.931) |
| Distant metastasis | *PDIA5* | 0.048 | 1.236 (1.091, 2.803) |
| Disease-free survival | *ABCA1*  *PDIA5* | 0.042  0.040 | 1.453 (1.199, 2.183)  1.443 (1.096, 2.177) |
| Disease-specific survival | *ABCA1*  *PDIA5* | 0.042  0.051 | 1.435 (1.314, 2.183)  1.522 (1.099, 2.343) |
| Overall survival | *ABCA1*  *PDIA5*  *RBM15B* | 0.015  0.006  0.024 | 2.370 (1.181, 4.762)  2.296 (1.266, 4.164)  2.370 (1.121, 5.000) |
