# Supplementary material for: MicroRNAs MiR-218, MiR-125b, and Let-7g Predict Prognosis in Patients with Oral Cavity Squamous Cell Carcinoma
Source: PLoS One. 2014 Jul 22;9(7):e102403. doi: 10.1371/journal.pone.0102403 (PMC4106832; doi:10.1371/journal.pone.0102403)
Supplement: Table S6 — Logistic regression analysis of clinical outcomes independently associated with the TP53-associated signatures. (DOC) [file pone.0102403.s007.doc]

**Table S6** Logistic regression analysis of clinical outcomes associated with the *TP53*-associated signatures

| **Event** | **Signature** | **P value** | **Odds ratio (95%CI)** |
| --- | --- | --- | --- |
| Neck control | *FZD6*  *TNFSF10*  *KAT2B*  *GUSB* | 0.048  0.054  0.025  0.037 | 2.372 (1.852, 6.603)  3.115 (1.471, 10.309)  5.155 (1.227, 21.656)  2.833 (1.065, 7.519) |
| Disease-free survival | *TNFSF10*  *NDUFB9* | 0.017  0.054 | 2.137 (1.148, 3.984)  1.441 (1.197, 2.151) |
| Disease-specific survival | *TNFSF10*  *NDUFB9* | 0.046  0.044 | 1.323 (1.117, 1.838)  1.478 (1.099, 2.200) |
| Overall survival | *NDUFB9*  *GUSB* | 0.01  0.036 | 1.891 (1.163, 3.077)  1.484 (1.027, 2.146) |
