# Supplementary material for: MicroRNAs MiR-218, MiR-125b, and Let-7g Predict Prognosis in Patients with Oral Cavity Squamous Cell Carcinoma
Source: PLoS One. 2014 Jul 22;9(7):e102403. doi: 10.1371/journal.pone.0102403 (PMC4106832; doi:10.1371/journal.pone.0102403)
Supplement: Table S7 — Logistic regression analysis of clinical outcomes associated with the miR-218, let-7g, and miR-125b in validation cohort. (DOC) [file pone.0102403.s008.doc]

**Table S7** Logistic regression analysis of clinical outcomes associated with the miR-218, let-7g, and miR-125b in validation cohort

| **Event** | **Predictor** | **P value** | **Odds ratio (95%CI)** |
| --- | --- | --- | --- |
| Disease-free survival | *miR-218* | 0.022 | 1.919 (1.096,3.336) |
| Disease-specific survival | *miR-218* | 0.016 | 1.988 (3.484,1.134) |
| Disease-free survival | *let-7g* | 0.025 | 2.688 (1.312,6.289) |
| Disease-specific survival | *let-7g* | 0.022 | 12.987(12.346. 43.478) |
| Disease-free survival | *miR-125b* | 0.013 | 1.98 (1.157, 3.391) |
| Disease-specific survival | *miR-125b* | 0.014 | 1.97 (1.15,3.387) |
