# Supplementary material for: MicroRNAs MiR-218, MiR-125b, and Let-7g Predict Prognosis in Patients with Oral Cavity Squamous Cell Carcinoma
Source: PLoS One. 2014 Jul 22;9(7):e102403. doi: 10.1371/journal.pone.0102403 (PMC4106832; doi:10.1371/journal.pone.0102403)
Supplement: Table S8 — Evidence derived from previous studies on the interactions between the network edges of the miRNA modulators. (DOC) [file pone.0102403.s009.doc]

**Table S8 Evidence derived from previous studies on the interactions between the network edges of the miRNA modulators**

| **Interaction** | **References** |
| --- | --- |
| mir-218 – *SP1* | [1] |
| let-7g – *MYC* | [2] |
| mir-125b – *TP53* | [3], [4], [5] |
| *SP1* – *ABCA1* | [6], [7] |
| *SP1* – *DDIT3* | [8], [9] |
| *SP1 – FAT1* | [10] |
| *SP1 – NDUFS8* | [11], [12] |
| *SP1 – EXT2* | [13] |
| *SP1 – GUSB* | [14] |
| *SP1 – TNFSF10* | [15], [16] |
| *SP1 – TNFRSF12A* | [17] |
| *MYC – ABCA1* | [18], [19] |
| *MYC – PDIA5* | [20], [21], [22] |
| *TP53 – FAT1* | [23] |
| *TP53 – NDUFB9* | [24] |
| *TP53 – GUSB* | [25], [26], [27] |
| *TP53 – TNFSF10* | [28] |

**References**

1. Ogawa T, Iizuka M, Sekiya Y, Yoshizato K, Ikeda K, et al. (2010) Suppression of type I collagen production by microRNA-29b in cultured human stellate cells. Biochem Biophys Res Commun 391: 316-321.

2. Lan FF, Wang H, Chen YC, Chan CY, Ng SS, et al. (2011) Hsa-let-7g inhibits proliferation of hepatocellular carcinoma cells by downregulation of c-Myc and upregulation of p16(INK4A). Int J Cancer 128: 319-331.

3. Le MT, Teh C, Shyh-Chang N, Xie H, Zhou B, et al. (2009) MicroRNA-125b is a novel negative regulator of p53. Genes Dev 23: 862-876.

4. Inui M, Martello G, Piccolo S (2010) MicroRNA control of signal transduction. Nat Rev Mol Cell Biol 11: 252-263.

5. Le MT, Shyh-Chang N, Khaw SL, Chin L, Teh C, et al. (2011) Conserved regulation of p53 network dosage by microRNA-125b occurs through evolving miRNA-target gene pairs. PLoS Genet 7: e1002242.

6. Thymiakou E, Zannis VI, Kardassis D (2007) Physical and functional interactions between liver X receptor/retinoid X receptor and Sp1 modulate the transcriptional induction of the human ATP binding cassette transporter A1 gene by oxysterols and retinoids. Biochemistry 46: 11473-11483.

7. Schmitz G, Langmann T (2005) Transcriptional regulatory networks in lipid metabolism control ABCA1 expression. Biochim Biophys Acta 1735: 1-19.

8. Reed BD, Charos AE, Szekely AM, Weissman SM, Snyder M (2008) Genome-wide occupancy of SREBP1 and its partners NFY and SP1 reveals novel functional roles and combinatorial regulation of distinct classes of genes. PLoS Genet 4: e1000133.

9. Gately DP, Howell SB (1996) Paclitaxel activation of the GADD153 promoter through a cellular injury response element containing an essential Sp1 binding site. J Biol Chem 271: 20588-20593.

10. Enquobahrie DA, Williams MA, Qiu C, Muhie SY, Slentz-Kesler K, et al. (2009) Early pregnancy peripheral blood gene expression and risk of preterm delivery: a nested case control study. BMC Pregnancy Childbirth 9: 56.

11. Lescuyer P, Martinez P, Lunardi J (2002) YY1 and Sp1 activate transcription of the human NDUFS8 gene encoding the mitochondrial complex I TYKY subunit. Biochim Biophys Acta 1574: 164-174.

12. de Sury R, Martinez P, Procaccio V, Lunardi J, Issartel JP (1998) Genomic structure of the human NDUFS8 gene coding for the iron-sulfur TYKY subunit of the mitochondrial NADH:ubiquinone oxidoreductase. Gene 215: 1-10.

13. Megy K, Audic S, Claverie JM (2002) Heart-specific genes revealed by expressed sequence tag (EST) sampling. Genome Biol 3: RESEARCH0074.

14. Tomatsu S, Orii KO, Islam MR, Shah GN, Grubb JH, et al. (2002) Methylation patterns of the human beta-glucuronidase gene locus: boundaries of methylation and general implications for frequent point mutations at CpG dinucleotides. Genomics 79: 363-375.

15. Chan J, Prado-Lourenco L, Khachigian LM, Bennett MR, Di Bartolo BA, et al. (2010) TRAIL promotes VSMC proliferation and neointima formation in a FGF-2-, Sp1 phosphorylation-, and NFkappaB-dependent manner. Circ Res 106: 1061-1071.

16. Nebbioso A, Clarke N, Voltz E, Germain E, Ambrosino C, et al. (2005) Tumor-selective action of HDAC inhibitors involves TRAIL induction in acute myeloid leukemia cells. Nat Med 11: 77-84.

17. Zellmer S, Schmidt-Heck W, Godoy P, Weng H, Meyer C, et al. (2010) Transcription factors ETF, E2F, and SP-1 are involved in cytokine-independent proliferation of murine hepatocytes. Hepatology 52: 2127-2136.

18. Santamarina-Fojo S, Peterson K, Knapper C, Qiu Y, Freeman L, et al. (2000) Complete genomic sequence of the human ABCA1 gene: analysis of the human and mouse ATP-binding cassette A promoter. Proc Natl Acad Sci U S A 97: 7987-7992.

19. Dong J, Sutor S, Jiang G, Cao Y, Asmann YW, et al. (2011) c-Myc regulates self-renewal in bronchoalveolar stem cells. PLoS One 6: e23707.

20. Zeller KI, Zhao X, Lee CW, Chiu KP, Yao F, et al. (2006) Global mapping of c-Myc binding sites and target gene networks in human B cells. Proc Natl Acad Sci U S A 103: 17834-17839.

21. Chen Y, Blackwell TW, Chen J, Gao J, Lee AW, et al. (2007) Integration of genome and chromatin structure with gene expression profiles to predict c-MYC recognition site binding and function. PLoS Comput Biol 3: e63.

22. Nishiyama A, Xin L, Sharov AA, Thomas M, Mowrer G, et al. (2009) Uncovering early response of gene regulatory networks in ESCs by systematic induction of transcription factors. Cell Stem Cell 5: 420-433.

23. Wei CL, Wu Q, Vega VB, Chiu KP, Ng P, et al. (2006) A global map of p53 transcription-factor binding sites in the human genome. Cell 124: 207-219.

24. Smeenk L, van Heeringen SJ, Koeppel M, van Driel MA, Bartels SJ, et al. (2008) Characterization of genome-wide p53-binding sites upon stress response. Nucleic Acids Res 36: 3639-3654.

25. Liu S, Mirza A, Wang L (2004) Generation of p53 target database via integration of microarray and global p53 DNA-binding site analysis. Methods Mol Biol 281: 33-54.

26. Wang L, Wu Q, Qiu P, Mirza A, McGuirk M, et al. (2001) Analyses of p53 target genes in the human genome by bioinformatic and microarray approaches. J Biol Chem 276: 43604-43610.

27. Wang J, Tian T (2010) Quantitative model for inferring dynamic regulation of the tumour suppressor gene p53. BMC Bioinformatics 11: 36.

28. Kuribayashi K, Krigsfeld G, Wang W, Xu J, Mayes PA, et al. (2008) TNFSF10 (TRAIL), a p53 target gene that mediates p53-dependent cell death. Cancer Biol Ther 7: 2034-2038.
