# Supplementary material for: MicroRNAs MiR-218, MiR-125b, and Let-7g Predict Prognosis in Patients with Oral Cavity Squamous Cell Carcinoma
Source: PLoS One. 2014 Jul 22;9(7):e102403. doi: 10.1371/journal.pone.0102403 (PMC4106832; doi:10.1371/journal.pone.0102403)
Supplement: Method S1 — Description of the sparse partial least squares regression. (DOC) [file pone.0102403.s010.doc]

**Sparse Partial Least Squares Regression (SPLS)**

Using a prediction matrix X and a response matrix Y, the SPLS model estimates the k^th^ column of the transformation matrix W to identify the latent variables that have the maximum covariance with the response variables. Moreover, it selects the optimal set of covariates with both *L_1_* and L2 penalty.


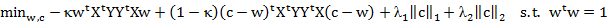


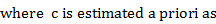


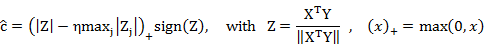


and κ and η are prespecified constants.
